# Feature Selection Framework for Improved UAV-Based Detection of *Solenopsis invicta* Mounds in Agricultural Landscapes

**DOI:** 10.3390/insects16080793

**Published:** 2025-07-31

**Authors:** Chun-Han Shih, Cheng-En Song, Su-Fen Wang, Chung-Chi Lin

**Affiliations:** 1 Department of Geography, National Changhua University of Education, Changhua 500, Taiwan; harry820902@gmail.com (C.-H.S.); sfwang@cc.ncue.edu.tw (S.-F.W.); 2 Department of Biology, National Changhua University of Education, Changhua 500, Taiwan; pths5000@gmail.com

**Keywords:** red imported fire ant (RIFA), UAV multispectral imagery, feature selection, image classification, invasive species detection

## Abstract

The red imported fire ant (RIFA; *Solenopsis invicta*) is an invasive species that severely threatens ecology, agriculture, and public health in Taiwan. In this study, the feasibility of applying multispectral imagery captured by unmanned aerial vehicles (UAVs) to detect red fire ant mounds was evaluated in Fenlin Township, Hualien, Taiwan. A DJI Phantom 4 multispectral drone collected reflectance in five bands (blue, green, red, red-edge, and near-infrared), derived indices (normalized difference vegetation index, NDVI, soil-adjusted vegetation index, SAVI, and photochemical pigment reflectance index, PPR), and textural features. According to analysis of variance F-scores and random forest recursive feature elimination, vegetation indices and spectral features (e.g., NDVI, NIR, SAVI, and PPR) were the most significant predictors of ecological characteristics such as vegetation density and soil visibility. Texture features exhibited moderate importance and the potential to capture intricate spatial patterns in nonlinear models. Despite limitations in the analytics, including trade-offs related to flight height and environmental variability, the study findings suggest that UAVs are an inexpensive, high-precision means of obtaining multispectral data for RIFA monitoring. These findings can be used to develop efficient mass-detection protocols for integrated pest control, with broader implications for invasive species monitoring.

## Simple Summary

Red imported fire ants are highly aggressive invasive insects that pose challenges to agriculture, ecosystems, and public health. Their nests, or mounds, are often difficult to spot in traditional ground surveys, which also tend to be time-consuming and labor-intensive. This study explored the possible application of drones equipped with special cameras that can detect variations in light reflected on the ground to locate fire ant mounds from above. The researchers examined characteristics such as plant health and soil visibility, discovering that certain color patterns in the images, particularly those related to plant cover and soil exposure, can reliably reveal mound locations. Additionally, various methods for processing the images were evaluated to improve accuracy. The innovative approach developed in this study can serve as a quick and cost-effective detection method for fire ant mounds over vast areas. This approach can assist farmers, local governments, and pest control workers in responding more swiftly and effectively to ant invasions, which may minimize their spread and impact.

## 1. Introduction

Invasive species have become a major global threat to agriculture, natural ecosystems, and public health [1,2,3]. Ants and other social insects are among the most successful invasive species worldwide. Numerous invasive ant species are present in Taiwan, including the yellow crazy ant (*Anoplolepis gracilipes*), the big-headed ant (*Pheidole megacephala*), and the red imported fire ant (RIFA) (*Solenopsis invicta*) [4]. Among these species, the RIFA is particularly notorious for its substantial ecological and economic impacts. Listed as one of the 100 of worst invasive species worldwide by the International Union for Conservation of Nature [5], the RIFA is a highly successful invader that causes an estimated US$ 5.01 billion in agricultural damages and control costs annually [6]. In addition to these substantial economic losses, the RIFA sting can pose severe health threats to humans, potentially causing life-threatening allergic reactions [7,8]. Genetic analyses have revealed that RIFA populations in Taoyuan, Taiwan originate from the southern United States [9]. Since this species’ introduction to Taiwan, RIFA has spread rapidly, with a 2020 study indicating that RIFA populations are progressively extending to additional counties [10].

Efficient detection and monitoring of invasive species are essential for timely management [11,12]. The RIFA has been present in Taiwan for an extended period, and several methods are commonly used to detect and monitor established colonies, including visual inspection, pitfall traps, and bait traps (e.g., oil baits). Some researchers have also used detection dogs to monitor RIFA and ant mounds, ensuring that no fire ants are present in designated areas, such as airports [13,14]. Government agencies and researchers continue to rely on these methods to survey large areas [15]. However, conventional techniques for detecting RIFA infestations are highly labor-dependent and require substantial human resources and field time, indicating a need for automated and efficient detection methods.

Effective detection methods are crucial in monitoring invasive species. Although conventional techniques can be applied to identify the presence of individual RIFAs, which are small, mobile, and often concealed, the application of these methods in comprehensive and widespread surveys remains challenging. By contrast, RIFA mounds are large and stationary, providing an excellent target for remote sensing to locate their presence in various landscapes.

In the remote sensing detection process, RIFA mounds are identified on the basis of their differentiation from other surface types according to key features, such as spectral and textural properties. This classification is achieved through feature selection, which involves identifying the most informative predictors to enable precise differentiation between mounds and their surroundings. The combination of classification with accurate geographic positioning enables reliable detection of RIFA mounds, providing highly precise information regarding mound locations. Remote sensing technology provides high-resolution multispectral imagery. This imagery enables the extraction of essential features and serves as a cornerstone for classification-driven detection frameworks.

At the landscape-to-regional scale, remote sensing has long supported ecological inventories and invasive species risk mapping. Researchers have integrated spectral information with soil brightness indices as well as Bayesian mixture models in medium-resolution satellite products, such as Landsat, to estimate habitat suitability for RIFA. This approach enables surveillance efforts to be concentrated on less than 20% of the total survey area, substantially improving resource efficiency relative to that achievable through traditional ground-based surveys [16]. Remote sensing products, including near-infrared (NIR) and thermal imagery, were also proven effective for large-scale detection of RIFA mounds, guiding eradication efforts and informing automated detection systems [17]. Nevertheless, satellite platforms remain limited by their decameter-scale pixels, cloud interference, and fixed revisit cycles, which complicate the tracking of small, fast-moving biological targets or short-lived habitat changes. Unmanned aerial vehicles (UAVs) overcome these limitations by offering flexible, high-resolution imaging.

UAVs can take off on demand, fly below cloud cover, and capture ultrahigh-resolution imagery, that supports real-time, minimally invasive surveys. Ecologists have investigated the utilization of UAV-mounted thermal-infrared sensors for the detection of insect emergence events, representing a prospective application of remote sensing in entomology [18]. Meanwhile, a similarity-based algorithm built on time-series normalized difference vegetation index (NDVI; index of canopy greenness and vigor) composites predicted Africanized honey bee migration in Texas with 62–63% accuracy [19]. In addition, multispectral imaging has matured into a key biological monitoring tool, providing band-specific insights into biochemical and physiological states [20]. Submeter multispectral UAV surveys previously distinguished RIFA mounds from surrounding soil on the basis of contrasts in the green (560 nm) and red-edge (730 nm) bands [21], supporting the feasibility of this method for RIFA detection.

The selection of appropriate features is critical for RIFA mound classification because feature selection directly affects classification accuracy by maximizing class separability and minimizing pattern recognition errors. This principle is particularly relevant for hyperspectral data, which can have numerous spectral bands. Studies of simulated hyperspectral data have demonstrated that when training samples are limited, the Hughes phenomenon emerges: classification accuracy initially increases with the number of spectral bands but declines after reaching a peak [22,23]. Effective feature selection mitigates this problem by enabling prioritization of the most discriminative features, such as vegetation indices or spectral bands, to improve classification performance [24]. Although UAV multispectral imagery contains a limited number of spectral bands, the variety of texture features in such imagery considerably expands the number of variables available that may be effective in improving detection or classification models, leading to a need for robust evaluation strategies to streamline feature selection for RIFA mound identification.

In this study, we introduced a classification method for RIFA mound detection. We used UAV multispectral imagery to effectively distinguish RIFA mounds from other surfaces and compared the results of pixel-level and object-level classifications, which represent two common analytical scales. Our approach combines analysis of variance (ANOVA), correlation analysis, random forests, and mutual information to identify the most informative features. These features, encompassing spectral, textural, and index-based data, are essential to improving detection accuracy. Furthermore, we assessed the effectiveness of spectral versus textural features and their influence on classification performance. This study aims to establish a robust framework for monitoring RIFA, thereby facilitating large-scale surveillance and integrated pest-management strategies for invasive ant species.

## 2. Materials and Methods

### 2.1. Study Area

**Figure 1:**
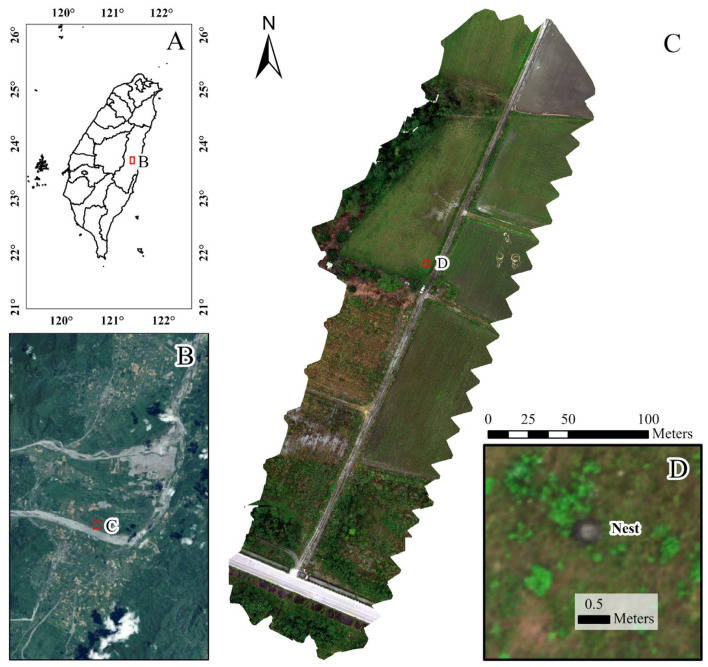
Study site location and UAV-derived orthomosaic imagery used for RIFA mound detection: (**A**) Red square on the map of Taiwan indicates the county of the study site; (**B**) red square on the Fenglin Township map indicates the location of the study site; (**C**) UAV-acquired orthomosaic image of the agricultural field, including surveyed area and landscape features; and (**D**) zoomed-in view highlighting a fire ant mound, with a spatial reference bar indicating the scale (0.5 m). All images were georeferenced through onboard GNSS and corrected with ground control points.

The study site is situated in Fenglin Township, Hualien County, Taiwan, and is characterized by a warm subtropical monsoon climate. The local annual average temperature is 23 °C, and the annual precipitation is 1850 mm. Field surveys conducted during 2020–2025 confirmed the presence of RIFA infestations in Fenglin Township. The study site primarily encompasses farmland, including agricultural fields, irrigation channels, and fallow land, that provides a suitable habitat for wildlife. The site has a low population density, offering conducive conditions for RIFA mounds and native wildlife. To comprehensively capture representative ecological variability, fire ant mounds were systematically located and selected across multiple ecological zones. We identified plots in our study area from previous survey reports on the basis of the presence of clearly visible RIFA mounds and suitability for UAV image acquisition. The study area (Figure 1) covered approximately 3 ha of flat terrain with an average elevation of 110 m. After active mounds were visually identified and confirmed, precise location data were recorded with a DJI D-RTK 2 Mobile Station (SZ DJI Technology Co., Ltd., Shenzhen, Guangdong, China) featuring a virtual reference station real-time kinematic (VRS-RTK) mode. After the investigation, the global navigation satellite system (GNSS) data were exported and examined for outliers or inaccurate readings.

### 2.2. Data Collection Methods

#### 2.2.1. UAV Specification

A Phantom 4 Multispectral drone (P4M; SZ DJI Technology Co., Ltd., Shenzhen, Guangdong, China) was used for data collection throughout the study. The system contains six cameras: five multispectral sensors (blue, green, red, red-edge, and NIR) and one red–green–blue (RGB) sensor, all with a focal length of 5.74 mm. Raw sensor images have dimensions of 1600 × 1300 pixels and a sensor size of 4.87 mm × 3.96 mm (1/2.9-inch complementary metal oxide semiconductor sensor). Irradiance data were collected through an onboard sunlight sensor, with postprocessing applied for conversion into usable reflectance values.

#### 2.2.2. Flight Parameters

To balance image clarity with survey efficiency, the drone was operated at 40 m above ground level. At this flight height, a of approximately 2.6 cm per pixel was achieved, which is an adequate resolution for the identification of small features, such as RIFA mounds without excessive coverage loss per flight.

#### 2.2.3. Ground Control Points

Eight ground control points were established systematically across the field, each marked with a 2 × 2 spray-painted checkerboard pattern. Coordinates were measured with a dual-frequency GNSS receiver under VRS-RTK mode. The horizontal and vertical accuracies were ±0.025 m and ±0.035 m, respectively. The same ground control points were later used in the photogrammetric process to improve the georeferencing accuracy of the final orthomosaic imagery.

#### 2.2.4. Sampling

This study focuses on visible ant mounds exceeding 10 cm in diameter. The field survey commences with an initial visual assessment of potential mounds to establish foundational familiarity with the site. Subsequently, we verify whether a mound is active by gently stimulating it through turning or probing the soil, thereby ensuring that only living mounds are selected for further marking and image analysis. For each confirmed mound, we recorded its centroid coordinates utilizing a dual-frequency GNSS receiver operating in VRS-RTK mode, which offers centimeter-level accuracy for subsequent region of interest (ROI) delineation within the UAV orthomosaics. Mounds with soil piles smaller than 10 cm or those only suspected of being ant mounds were excluded to prevent misclassification and to maintain focus on mature mounds of ecological significance. This method resulted in more precise spectral data and provided a clearer understanding of the distribution of RIFA colonies.

### 2.3. Image Processing and Analysis

#### 2.3.1. Reflectance Conversion

Raw data (digital number; DN) from the DJI P4M drone were converted to reflectance values through the P4M tool [25,26], an open-source software for radiometric correction that was included with the drone. A downwelling light sensor provided real-time data during conversion, including exposure time and irradiance measurement, to recalculate the sunlight intensity while the UAV was in flight. Notably, rather than a reflectance panel or any predefined reflectance targets, the P4M workflow involved the use of a simplified, widely used, UAV-based remote sensing strategy. For instance, image processing standards by MicaSense suggest a constant direct-to-diffuse irradiance ratio of 6.0 for clear-sky conditions [26]. Although the continuous ratio may not reflect complete atmospheric variability, it is an operational compromise, providing adequate calibration accuracy and simplifying field-based UAV operations.

#### 2.3.2. Orthomosaic Generation and Layer Stacking

**Figure 2:**
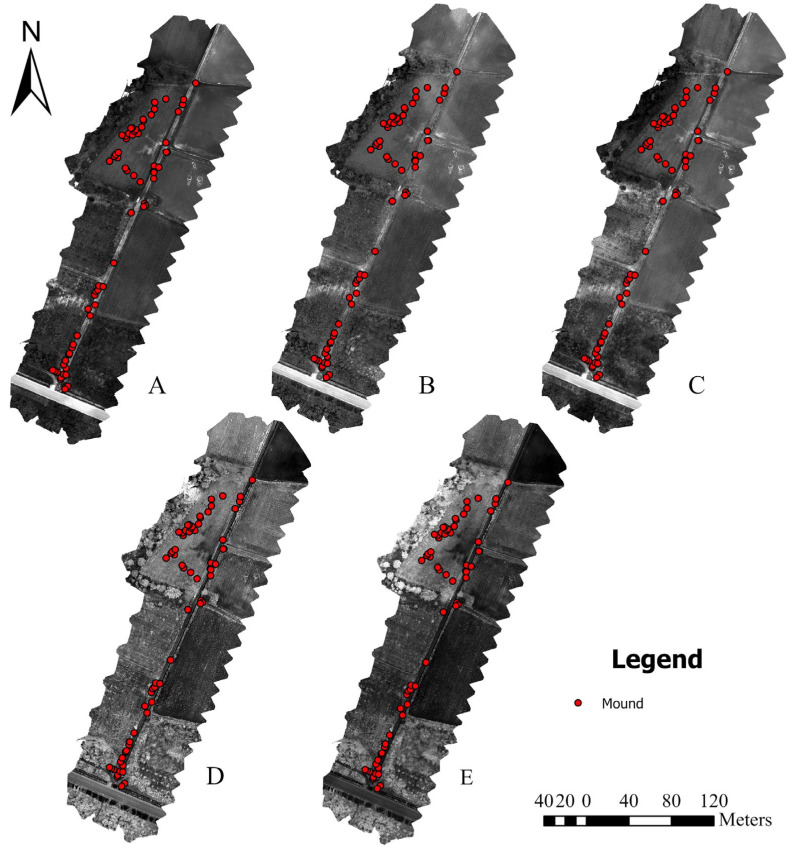
Orthomosaic maps of the study area across five spectral bands: (**A**) blue band (450 nm), (**B**) green band (560 nm); (**C**) NIR band (840 nm), (**D**) red-edge band (730 nm), and (**E**) red band (650 nm). Red dots indicate RIFA mound locations. Scale bars and north arrows are included for spatial reference.

After conversion, individual images were processed with Pix4D (Pix4D SA, Switzerland) to create a multispectral orthomosaic for each spectral band (blue, green, red, red-edge, and NIR) (Figure 2). These orthomosaics were stacked to produce a single composite image with five spectral layers. To extract more informative features from the imagery, vegetation indices and texture features were computed with reference to the orthomosaic image. Vegetation indices, typically derived from multispectral bands, involve leveraging the reflective properties of vegetation to enhance the distinction of vegetative or green elements. Texture analysis can be used to quantify the spatial distribution of pixel gray levels within an image, characterizing coarseness, smoothness, regularity, and other key properties as functions of spatial variation in pixel intensity [27]. ENVI 5.6 software was used to calculate three vegetation indices: the NDVI (index of canopy greenness and vigor), soil-adjusted vegetation index (SAVI; NDVI variant corrected for soil background, reliable in sparse vegetation), and photochemical pigment reflectance index (PPR; chlorophyll ratios for plant health via reflectance). Texture features were also calculated on the basis of the gray-level co-occurrence matrix (GLCM), which includes homogeneity, contrast, dissimilarity, entropy, second moment, and correlation (Table 1). The initial feature set comprised both raw spectral bands and derived indices.

#### 2.3.3. Spatial Scale of Analysis: Pixel-Level Versus Object Representation

We analyzed the spectral and textural characteristics of RIFA mounds across two scales: the pixel level and the object level. These scales represent distinct concepts in remote sensing analysis. At the pixel level, each image pixel is considered an individual unit, enabling analysis at a fine scale and with high sensitivity to spectral anomalies. The internal heterogeneity of RIFA mounds or the spectral contrast in the background must be analyzed. At the object level, each RIFA mound is treated as a homogeneous unit. The two datasets were compared to evaluate the significance of the features across varying spatial scales, enabling us to assess RIFA mounds either as independent objects or at the pixel level and to treat them as stabilized features across a spatial scale for future classification.

**Figure 3:**
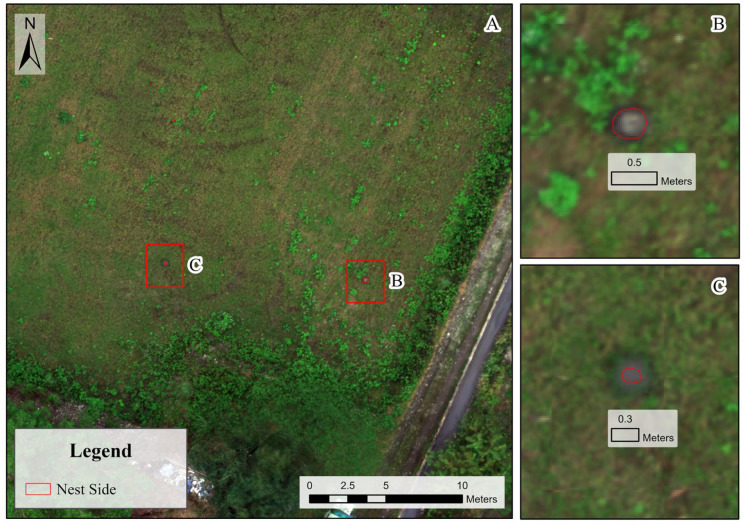
Study site. (**A**) Close-up of part of the study site. (**B**) Aerial view of RIFA mound. Scale indicates the mound’s size to be 0.5 m. (**C**) Additional view of RIFA mound.

For each RIFA mound observed in the field, we marked a region of interest (ROI) in ArcGIS using UAV imagery (Figure 3), based on data collected in the field. For each ROI, the mean reflectance values of the spectral, index, and texture features were extracted to capture the characteristics of the RIFA mounds. Under this approach, RIFA mounds are viewed as an independent biological feature. This dual-scale approach supports further exploration of the role and implications of spatial analysis in UAV-based ecological monitoring.

### 2.4. Feature Selection and Redundancy Removal Strategy

First, to construct a robust feature set for classification, we implemented a dual-pathway framework integrating both linear and nonlinear perspectives. Specifically, two complementary routes were employed: (i) a linear route that utilizes ANOVA F-score ranking, followed by the elimination of redundant variables through Pearson filtering (|r| > 0.7), thereby preserving the most informative and independent linear features; (ii) a nonlinear route that applies random forest RFE ranking, subsequently pruning redundant variables via maximal information coefficient (MIC) filtering (>0.7), thus capturing multivariate and nonlinear dependencies. The integration of variables retained by these two routes yields a compact yet information-rich feature set that leverages the advantages of both linear and nonlinear perspectives. In the linear pathway, we applied ANOVA F-scores to assess the discriminative power of each feature, successfully prioritizing features that clearly distinguished RIFA mounds from other surface types. Subsequently, we conducted Pearson correlation analysis to identify highly correlated feature pairs (|r| > 0.7) and strategically removed the feature with the lower F-score to minimize redundancy [28]. In the nonlinear feature-selection pathway, we applied recursive feature elimination (RFE) with a random forest estimator to compute feature importance scores and obtain a stable global ranking; the intermediate “elimination order” generated through RFE was not used, and no variables were discarded at this stage. We then calculated the MIC for each pair of spectral, vegetation index, and texture features with the minepy package [29]. Pairs with an MIC value greater than 0.7 were considered highly correlated; within each such pair, the feature with the lower RFE importance score was removed to minimize redundancy while preserving discriminative power. We selected the 0.7 threshold in accordance with the “strong-correlation” convention widely adopted in remote sensing and environmental machine-learning studies [30]. This threshold is conceptually consistent with the traditional |r| > 0.7 rule used for Pearson-based filtering. MIC captures both linear and nonlinear dependencies, offering a robust filter for dimensionality reduction. This capability is especially valuable for high-dimensional remote sensing datasets that combine multispectral bands with granulometric-based texture layers, in which intricate interfeature redundancies are common [31]. The combination of ANOVA F-score, Pearson correlation, random forest RFE, and MIC techniques ensured that the selected features were both predictive and nonredundant across linear and nonlinear paradigms. Linear pathways were integrated with nonlinear pathways through the combination of retained features and elimination of duplicates, producing a cohesive feature set. We used the scikit-learn tool in Python 3.9.21 to analyze ANOVA F-scores and RFE and the pandas tool for Pearson correlation analysis of both object-level and pixel-level datasets, which comprised 38 spectral, index, and texture features. The final features in both datasets underwent rigorous validation to confirm their consistency across multiple spatial scales and adaptability to various modeling frameworks.

Second, to further evaluate feature performance across different methods and spatial scales, we combined linear and nonlinear approaches with object-level and pixel-level datasets to create four comparison groups: linear/pixel-level, linear/object-level, nonlinear/pixel-level, and nonlinear/object-level groups. We compared feature retention counts and the stability of key rankings (e.g., cross-validation coefficient of variation) to assess the consistency, stability, and predictive power of spectral, index, and texture features. This dual-scale approach validated the robustness of feature selection and further highlighted the critical role of spatial resolution in UAV-based ecological monitoring, providing a multidimensional foundation for subsequent classification tasks.

Third, to visually interpret the comparative results, we constructed a normalized heatmap summarizing feature importance in all four groups. Each importance score, or F-score, was initially standardized within its respective method and spatial scale to facilitate cross-contextual comparisons. Furthermore, we quantified the frequency with which each feature was retained across models, treating this retention frequency as a proxy for stability and reliability. This multimethod approach represents a robust framework for the selection of informative and nonredundant features, adapted to both modeling complexity and spatial resolution. The framework provided a reliable foundation for downstream classification and ecological inference, ensuring that the selected features were both statistically sound and ecologically interpretable.

## 3. Results

### 3.1. Analysis of Multispectral Characteristics

**Figure 4:**
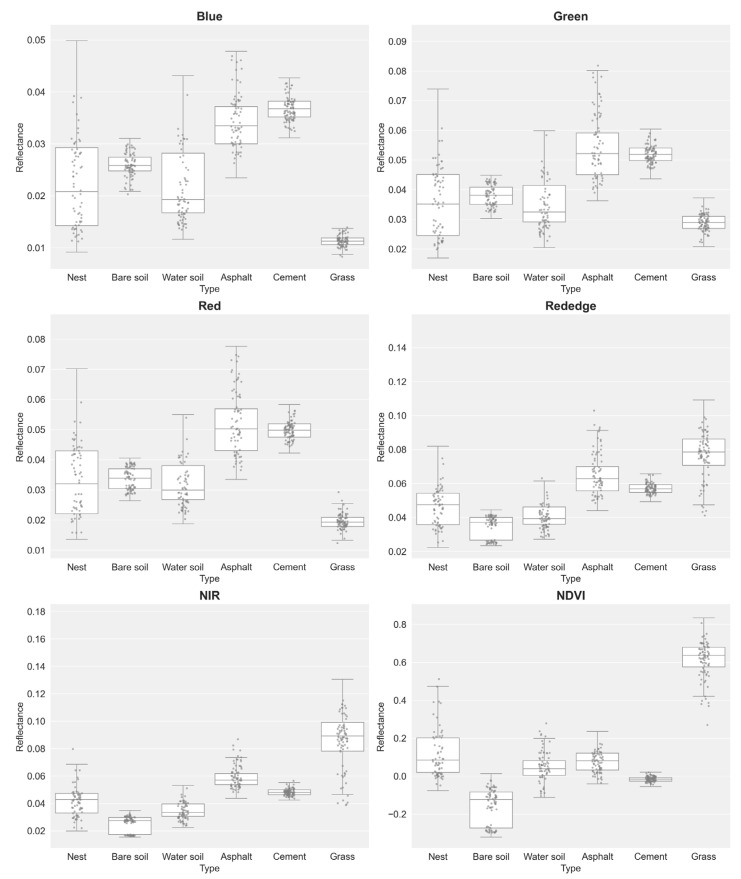
Box plots of pixel-level reflectance value distributions for different surface types across five spectral bands and the NDVI: Type 1: RIFA mounds, Type 2: bare soil, Type 3: water-containing soil, Type 4: asphalt, Type 5: cement, and Type 6: grass.

**Figure 5:**
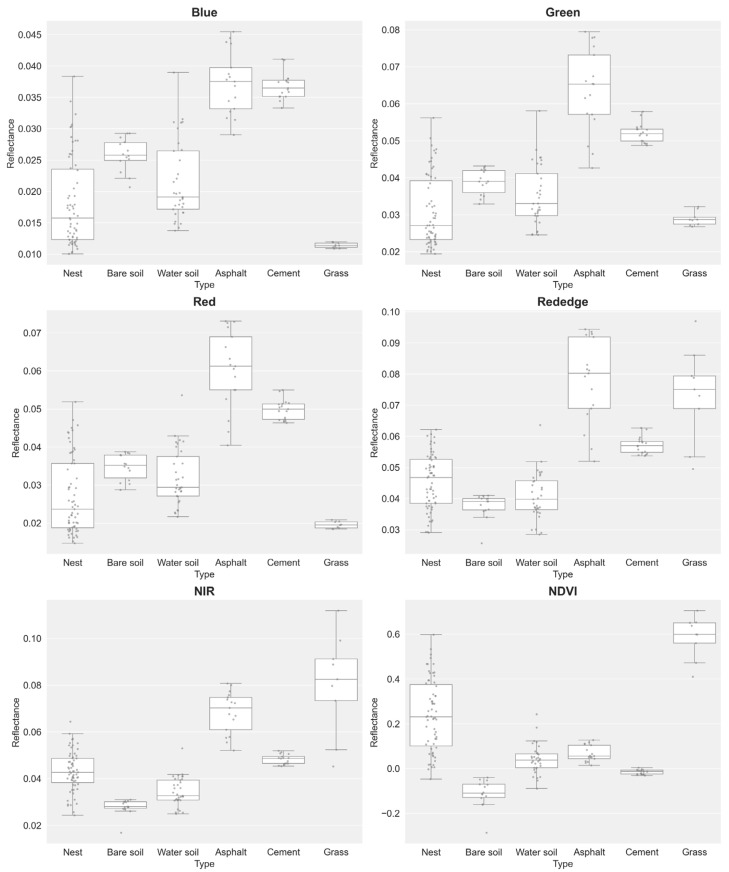
Box plots of object-level reflectance value distributions for different surface types across five spectral bands and the NDVI: Type 1: RIFA mounds, Type 2: bare soil, Type 3: water-containing soil, Type 4: asphalt, Type 5: cement, and Type 6: grass.

Analysis of multispectral data (pixel-level and object-level datasets; Figure 4 and Figure 5) revealed distinct reflectance patterns across surface types. RIFA mounds exhibited low reflectance with high variability in RGB bands. At the pixel level, RIFA mounds had median reflectance values of 0.012 (blue), 0.023 (green), and 0.018 (red); at the object level, these values were 0.015 (blue), 0.025 (green), and 0.020 (red). By contrast, the RGB bands of asphalt surfaces exhibited markedly higher reflectance, with object-level median values of 0.030 (blue), 0.065 (green), and 0.061 (red). Vegetation-covered areas, such as grass, exhibited the lowest RGB band reflectance but the highest NIR and red-edge band reflectance, consistent with typical spectral signatures for vegetation.

In the NIR band, the median reflectance values for RIFA mounds were 0.0428 (pixel level) and 0.0426 (object level), which are lower than those of asphalt (pixel level: 0.0569; object level: 0.0702), cement (both levels: 0.048), and grass (pixel level: 0.089; object level: 0.082). In the red-edge band, asphalt exhibited a distinct reflectance value at the object level, though specific data were not obtained.

The NDVI (index of canopy greenness and vigor) values clearly differed between surface types, effectively differentiating vegetative from nonvegetative surfaces. Grass exhibited the highest median NDVI values at 0.636 (pixel level) and 0.599 (object level), reflecting dense vegetation cover. For RIFA mounds, positive but low NDVI values of 0.085 (pixel level) and 0.231 (object level) were recorded, likely indicating sparse vegetation or mixed surfaces. Bare soil corresponded to negative NDVI values of −0.123 (pixel level) and −0.111 (object level), and cement had near-zero values of −0.016 (pixel level) and −0.015 (object level), which is characteristic of nonvegetated surfaces.

These results confirm the robust capability of the NDVI to distinguish vegetation from impervious or bare surfaces. However, the discriminative power of the NDVI was limited with respect to separating RIFA mounds from other low-reflectance, nonvegetated surfaces, such as cement, particularly at the pixel level, at which RIFA mounds (0.085) and cement (−0.016) had relatively close values. This limitation was less pronounced at the object level (0.231 vs. −0.015) and did not apply to bare soil, which was consistently distinguishable. The reduced discriminative power likely stems from overlapping spectral signatures in the visible and NIR bands.

### 3.2. Feature Selection for Pixel-Level Data

**Figure 6:**
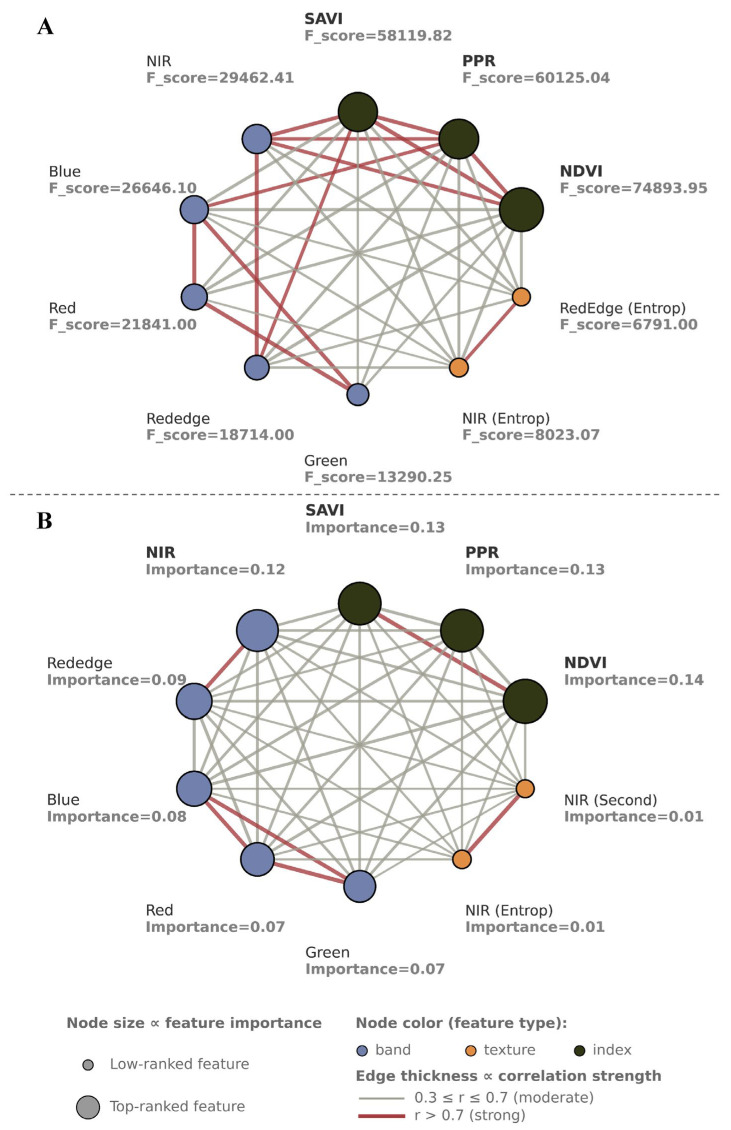
(**A**) ANOVA F-scores (linear model) and (**B**) feature importance (nonlinear model) for pixel-level features.

We performed feature extraction on the pixel-level data to examine the significance of spectral parameters at a finer spatial resolution. Linear analysis of ANOVA F-scores (Figure 6A) was conducted to determine the ability to discriminate between RIFA mounds and the surrounding environment for each feature. After removing highly collinear features (Pearson |r| > 0.7; e.g., PPR, SAVI, NIR, and Red), the NDVI, blue, and red-edge bands consistently exhibited the largest F-score across the dataset (Appendix A.1), implying that each of these bands had a strong linear relationship to the class goal. These bands could effectively separate pixels assigned to RIFA mounds from other pixels. Other features, such as the texture of NIR (entropy) and red-edge (entropy) bands, had moderate discriminative capacity, improving model performance. Highly intercorrelated features, especially those of correlation coefficient–based features, had lower F-scores and were therefore less informative.

For nonlinear features (Figure 6B), after removing highly collinear features (MIC > 0.7; e.g., SAVI), the NDVI, PPR, and NIR bands were generally the top-ranked features (Appendix A.2), reflecting the linear analysis findings with the addition of moderately important and texture features, such as NIR dissimilarity. Features with low importance values and correlation coefficients were considered less informative and removed to eliminate feature overlapping and improve classifier stability.

### 3.3. Feature Selection for Object-Level Data

**Figure 7:**
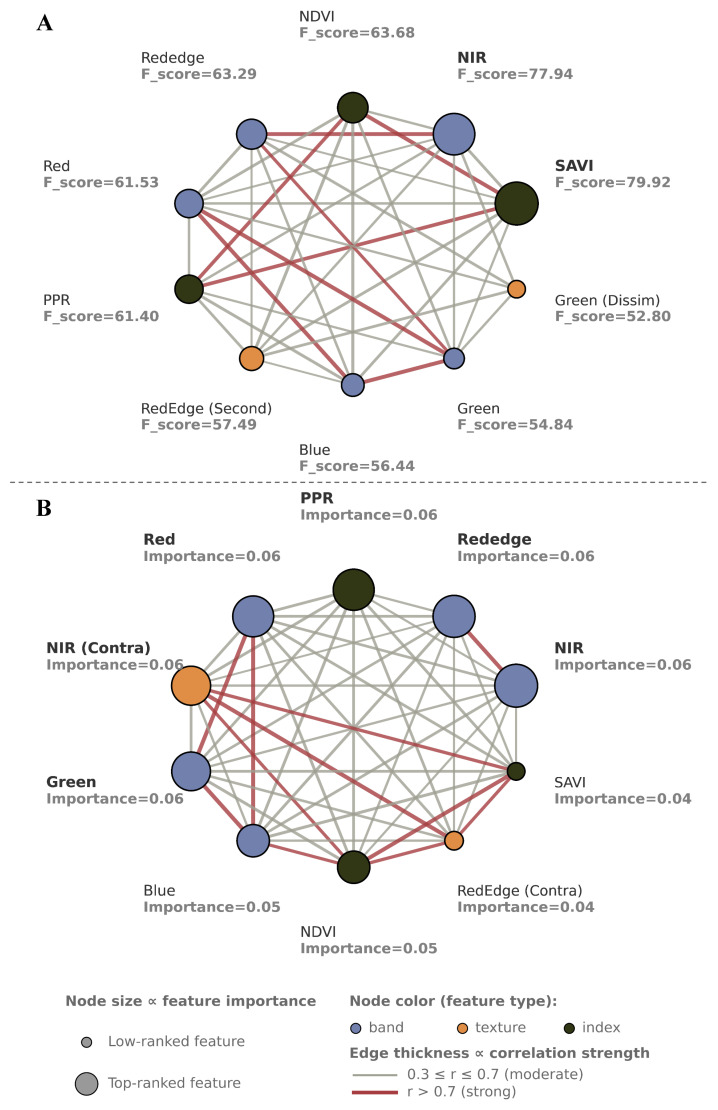
(**A**) ANOVA F-scores (linear model) and (**B**) object-level feature importance (nonlinear model) for object-level features.

Multispectral images have traditionally been classified on the basis of the spectral differences between various land cover types. With greater spatial resolution, pixel-level classification is prone to the salt-and-pepper effect, creating classification noise. Object-oriented classification that accounts for spatial correlation has emerged as an alternative approach. In this study, we first segmented the multispectral orthomosaic image into objects on the basis of the homogeneity of adjacent pixels. ROIs were then selected from these objects for object-level feature selection analysis. We evaluated the relationships between object features and the target classification according to linear ANOVA F-scores (Figure 7A). After removing highly collinear feature (Pearson |r| > 0.7; e.g., NDVI and rededge), the highest F-scores were observed for the SAVI, NIR band, and red band (Appendix A.3), indicating these features had strong linear ability to distinguish RIFA mounds from other surface types. Other features, including red-edge (second moment), green dissimilarity, and blue secondary features, were considered moderately informative, reflecting a potential contribution to improved classification accuracy.

Nonlinear feature selection was also performed to complement the linear analysis. Random forest RFE was applied to capture possible nonlinear interactions between features. In the RFE analysis (Figure 7B), after removing highly collinear feature (MIC > 0.7; e.g., Rededge)**,** the most important features were the NIR band, PPR, and red band (Appendix A.4), among which NIR had the highest importance. Additionally, textural and contextual information, such as NIR (dissimilarity) and green (contrast) bands, were moderately informative, highlighting their importance in retaining spatial heterogeneity related to ant mounds. Features with persistently low importance scores, most noticeably the correlation coefficient features, were deemed less informative and removed to minimize redundancy and model overfitting.

### 3.4. Comparison of Feature Selection Scales and Methods

**Figure 8:**
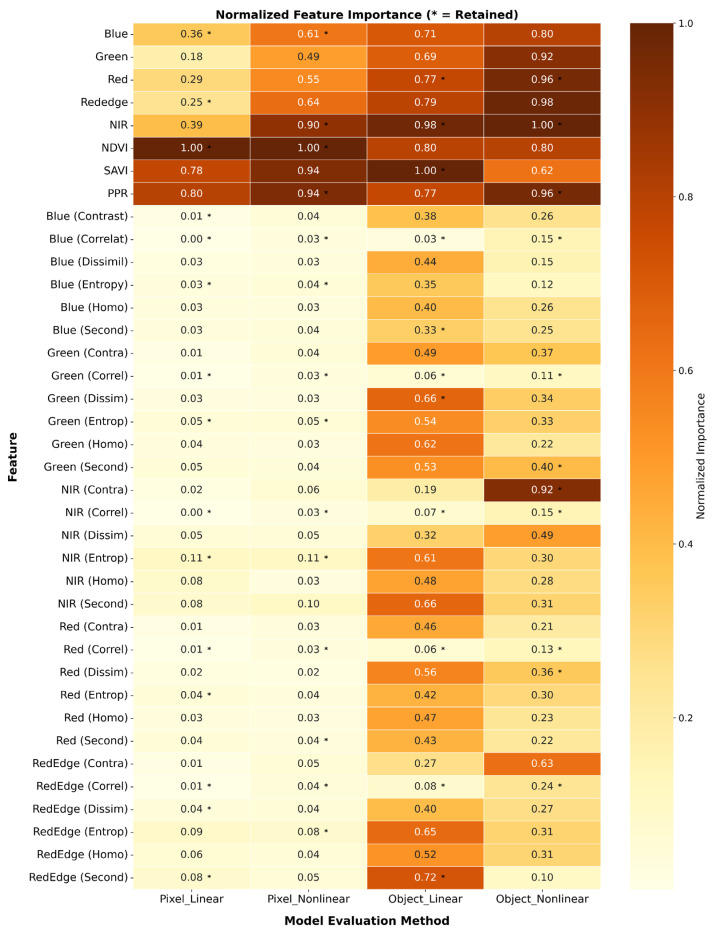
Normalized feature importance heatmap across four methodology configurations, with retained features marked. Spectral and index features exhibited consistently high importance. Asterisk (*) indicates whether a feature was retained under a given method.

To evaluate feature performance across spatial scales and model types, we normalized the feature importance scores calculated under the four feature selection methods (object-level linear, object-level RFE nonlinear, pixel-level linear, and pixel-level nonlinear) and plotted them on a heat map (Figure 8). The vegetation indices and spectral bands attained generally high scores and were retained in most models, whereas the performance of the texture features was relatively weak.

Among all models, the NDVI and NIR band were the features with the highest normalized scores. The NDVI was retained under two out of four methods, and the NIR band was retained under three methods. Additionally, the PPR (vegetation index; chlorophyll ratios for plant health via reflectance) was retained under two methods (object-level nonlinear and pixel-level nonlinear). Although the SAVI was ranked highly under certain methods, it was retained only once, primarily because of its high collinearity with the NDVI, leading to its exclusion during the removal of redundant features. Table 2 provides a summary of the top 10 features according to average importance across the four methods after redundant features were eliminated. NDVI ranked first in both pixel-level methods, and the NIR band ranked first in one object-level method. Other features, including the NIR (entropy), red-edge (second moment), and green (entropy) bands, also appear multiple times in the top 10.

**Figure 9:**
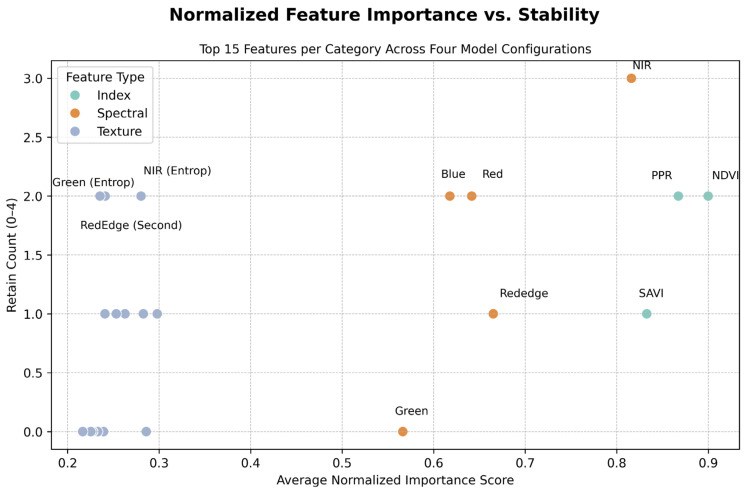
Scatterplot illustrating the relationship between the frequency of feature retention (y-axis) and the average normalized importance score (x-axis) for the top 15 features across four methods, comprising object-level and pixel-level approaches incorporating ANOVA F-score and RFE. Importance scores were normalized separately within each methodological category and then averaged.

Figure 9 further illustrates the average importance of each feature alongside the frequency of its retention across the four methods. The NDVI and NIR features are positioned in the upper right quadrant, signifying their high importance and retention frequency. The PPR feature is also located in this quadrant and was retained under two of the methods. The majority of the texture features are positioned in the lower left quadrant of the figure, corresponding to low scores and retention frequencies. Among these features, only the NIR (entropy) and green (entropy) bands were retained under pixel-level methods.

In summary, the vegetation indices and NIR spectral features exhibited notable importance and retention stability across methods and scales; conversely, the selection frequency and scoring of texture features was comparatively low.

## 4. Discussion

### 4.1. Discriminative Strength Across Feature Types

The retention of vegetation indices in several models underscores their effectiveness in RIFA mound detection, a finding consistent with those of previous studies [32,33] demonstrating their crucial role in distinguishing ant mounds. These features are capable of reflecting both vegetation stress and soil exposure, both of which change in response to the presence of RIFA mounds.

In this study, texture features did not perform well in linear analysis methods. However, in nonlinear analyses, the NIR (entropy) and green (entropy) band texture features demonstrated moderate importance. This finding suggests that advanced nonlinear methods can capture complex spatial patterns that simpler linear methods may fail to detect, supporting prior evidence that nonlinear learning models better apply object-level texture features [34,35]. Nevertheless, linear methods may be more appropriate in other circumstances, such as when the sample size is small. Our observations underscore the complexity and sensitivity of methodological performance. Results can differ according to dataset properties, feature extraction methods, and classifier configurations [36].

Overall, vegetation indices and NIR bands were identified as robust features for RIFA mound classification. Although texture features are less robust, they may be useful when complex methods are applied. Our feature selection method can help researchers to clearly understand which features are useful. In future studies, scholars should explore GLCM and granulometric texture, given their reported stability and resistance to edge effects in land cover classification [31].

### 4.2. Feature Stability and Importance Across Methods

Our findings, summarized in Figure 9, demonstrate that vegetation indices (NDVI, SAVI, and PPR) and the NIR band were consistently top-ranked in both ANOVA and random forest RFE analyses, highlighting the effectiveness of these features in detecting ecological signals in RIFA mound monitoring, such as low vegetation cover and exposed soil [37]. Notably, the retention of SAVI was limited under certain methods because of its high collinearity with NDVI, highlighting a need for redundancy removal in feature selection. These results underscore the robustness of NDVI (index of canopy greenness and vigor) and NIR features for ecological monitoring of invasive species, aligning with those of prior studies on ant mound detection [21]. The performance of these features corresponds to previously observed environmental conditions surrounding RIFA mounds, including minimal vegetation stress and soil exposure, which can be assessed according to changes in reflectance, especially through soil-normalized indices such as the SAVI (NDVI variant corrected for soil background, reliable in sparse vegetation) [38]. Similar remote sensing research has effectively identified the ecological effects of ants, including the detection of leaf-cutting ant mounds in teak plantations and measurement of defoliation by *Atta* ants in *Eucalyptus* plantations, supporting the valid application of these indices in ant mound monitoring [32,33].

Texture features were less informative in linear models but more useful in nonlinear models, including the random forest approach. This finding reflects the utility of textural features in assessments of complex spatial patterns. Similarly, Kupidura [31] improved land use mapping with satellite data, in which texture features effectively supplemented vegetation indices in measurements of the spatial heterogeneity. Although that study addressed generic landscape classes rather than RIFA mounds, the observed enhancement in accuracy reinforces the concept that texture can serve as a valuable complement to vegetation indices when delineating small, heterogeneous surface features such as fire ant mounds. Comparable improvements in accuracy resulting from the integration of texture and spectral data have been documented in UAV imagery applications for urban-vegetation mapping and other high-resolution ecological targets.

Although several features exhibited low variable importance in our analyses, permutation- and impurity-based studies have demonstrated that such variables may be retained because of their interactions with higher-ranked features, despite the variables not providing substantial marginal information [39,40]. Additionally, research indicates that the selection of approximately 10 optimal features improves model stability and performance in multispectral remote sensing, supporting our choice to limit the feature set size [24]. Consequently, our approach to feature selection balanced numerical significance with ecological validity, with intentional exclusion of variables that served as location proxies to prevent spatial overfitting. This approach is consistent with the principles of spatial variable selection discussed by Meyer et al. [41]. Our results reflect those of prior UAV analyses, in which NIR (and derived indices such as NDVI/SAVI) emerged as the most reliable indicators of *S. invicta* mounds [21]. Although texture descriptors provide limited value in linear frameworks, they can effectively capture higher-order spatial patterns in nonlinear approaches, as reported by Kupidura [31].

These results demonstrate that multispectral UAV-based monitoring systems for ant mounds must incorporate evaluation of feature types. Tailored evaluation is critical for the optimization of remote sensing applications, as has been reported in studies regarding machine learning-based forest cover classification and invasive species detection [11,34], underscoring the role of feature selection in refining UAV-based systems targeting RIFA mounds.

The NIR, NDVI, SAVI, and PPR were consistently among the most informative features across both linear (ANOVA) and nonlinear (RFE) frameworks. These features effectively capture ecological signals characteristic of RIFA mounds, such as reduced vegetative cover and exposed soil surfaces. Although we did not construct a classification model, the robustness and discriminative capacity of these features suggest their considerable potential for future automated RIFA mound detection. Previous research also highlighted the spectral contrast in the red and NIR bands [21], supporting their inclusion in future classification frameworks, although the model performance of these features requires empirical validation.

These findings provide a solid foundation for future feature-informed model development, particularly in terms of ecological and management implications. This framework not only reveals the impacts of fire ant mounds on ecosystems, for example, by capturing vegetation stress and soil exposure through NDVI and SAVI, thereby evaluating potential disruptions to biodiversity [42], but also offers practical applications in management. Specifically, this approach can be utilized for real-time field monitoring: UAVs are capable of processing multispectral images instantaneously, selecting critical features to generate mound heatmaps, and transmitting these data via mobile applications to on-site personnel for verification, thereby significantly reducing survey resources, with estimated time savings [43]. Moreover, this framework can be incorporated into pest alert systems, including GIS platforms, to automatically trigger alerts when mound density surpasses predefined thresholds, thus facilitating early intervention.

### 4.3. Research Limitations

This study demonstrated the potential of UAVs with multispectral sensors for RIFA mound identification. Nonetheless, several technical challenges persist. The flight altitude plays a particularly critical role in the clarity of image capture. Thus, achieving a precise balance between UAV flight altitude, image resolution, and survey coverage is essential. Higher altitudes reduce spatial resolution, which improves acquisition efficiency but may produce fuzzy images of mound objects, limiting detection. However, lower altitudes constrain coverage and increase operational risks near obstacles. Thus, achieving a balance between operational simplicity and sufficient spatial resolution is a major challenge within this research domain.

UAVs capture multispectral images, providing a cost-effective and flexible alternative platform for small-scale surveys in regions with intensive land use, such as Taiwan. Moreover, UAVs can easily adapt to diverse terrains and environments (e.g., riverbanks and dense shrublands), enabling swift deployment and retrieval. These qualities render UAVs ideal for small-scale, high-precision monitoring tasks. In Taiwan, UAVs have already been applied in spectral detection of agricultural diseases [44]. Although this method lacks precision for invasive species monitoring, the technical framework established herein provides a foundation for efficiently detecting RIFA invasions.

Although UAV-based multispectral imaging has demonstrated considerable potential for RIFA mound detection, our findings are not sufficiently robust to be considered definitive. Environmental factors, such as cloud cover and solar elevation, can substantially affect the quality of multispectral imagery, which can lead to unreliable reflectance measurements.

In addition to these environmental challenges, the framework in this study may lead to misclassifications in feature selection, such as overlaps in spectral signatures between ant mounds and similar soil features (e.g., bare soil or vegetation residues). Furthermore, seasonal variations (e.g., fluctuations in soil moisture due to rainfall or vegetation growth affecting mound visibility) may reduce detection accuracy, particularly impacting the performance of NIR and texture features. In pest alert systems, misinterpretation can lead to unnecessary treatments, causing disturbances to non-target species. To solve these issues, future work can integrate advanced machine learning algorithms (e.g., CNN models) with additional bands (e.g., short-wave infrared) or use multi-seasonal datasets for model calibration, along with time-series analysis to capture dynamic changes [45,46].

Despite these challenges, UAV technology is advantageous because it offers fast screening, shortening the time and labor required for large-scale field investigations and improving operational efficiency. To address the challenges associated with UAVs and improve data quality, we propose the following improvements.

First, reflectance calibration should be conducted. Visual reference values for reflectance correction can be derived through imaging standard reflectance panels before and after every flight. However, a study [47] indicated that panel-based calibration may be affected by solar radiation changes during flight, especially at sunrise or sunset. To solve this problem, irradiance-sensors can be used to achieve more stable reflectance outputs under varying illumination conditions, ensuring uniformity across times of day and environments. Fixed-reflectance materials (e.g., calibration boards of known spectral properties) at study locations can assist in accurate calibration to facilitate precise and reliable data collection.

Second, reflectance conversion must be improved to ensure data consistency across varying environmental contexts. Daniels et al. [48] demonstrated the accuracy of the multiple-radiometric-reference-target empirical line approach in UAV-based multispectral imagery. This approach can increase the robustness of UAV-based multispectral imagery and enable more routine use of UAV-based methods in RIFA surveys and other ecological monitoring.

The challenges observed herein align with broader concerns in remote sensing, particularly in hyperspectral data collection. Hughes [22] used simulated hyperspectral data to demonstrate the Hughes effect. When training samples are limited, classification accuracy first increases with the number of spectral bands, but declines after reaching a peak. This finding highlights a need for dimensionality reduction in hyperspectral applications. Common approaches to dimensionality reduction include feature selection, through which key bands that retain critical spectral characteristics are identified, and feature extraction, in which original features are transformed or combined into a new, reduced set. Although more features can theoretically improve classification accuracy, excessive features in limited training datasets may actually reduce classification performance (Hughes effect). This phenomenon implies that selection of essential features is necessary in studies featuring high-dimensional hyperspectral data [49]. We observed that hyperspectral cameras offer finer spectral resolution, improving the detection of subtle biochemical and physiological changes in vegetation but requiring complex data processing and larger training datasets to mitigate the Hughes effect. These techniques can be integrated into multispectral and hyperspectral UAV workflows to improve classification accuracy and model efficiency for RIFA detection, particularly in complex environments.

In addition to technical challenges, this study is limited by its scope. As preparatory work for the development of a detection model for RIFA mounds, this study was conducted in Fenglin Township, Hualien, Taiwan, covering a limited sample of identified mounds. The study site may not fully represent Taiwan’s diverse environmental conditions, which include urban areas, forested regions, and varied climatic zones. The subtropical monsoon climate and agricultural landscape of Fenglin likely influence the spectral and textural signatures of RIFA mounds, potentially restricting the applicability of the features identified in this study (NDVI, NIR, SAVI, and PPR) in other contexts. Additionally, we performed reflectance calibration with the P4M tool to mitigate environmental variability by standardizing multispectral data. However, the generalizability of these features across diverse regions remains untested. To increase the robustness and applicability of the detection framework, future studies with a larger sample size encompassing multiple regions with diverse topography, soil types, and climates should be conducted. Ground-based surveys, such as pitfall traps or visual inspections, could be integrated into UAV-based approaches to validate these results, improving feature reliability across heterogeneous landscapes.

### 4.4. Comparison of Detection Approaches: Deep Learning and Feature Selection Models

Deep learning models that incorporate convolutional spatial features can effectively detect ant mounds. This is especially true when they are trained on high-resolution imagery and deployed within a detection system [50,51]. The YOLO model, which is based on convolutional neural networks (CNNs), enables extraction of high-level spatial features that are particularly beneficial in areas characterized by high mound density or visually distinct landscapes.

Instead of CNNs, we employed a spectral-feature classification paradigm. Our approach emphasizes extraction of ecological signals rather than spatial pattern recognition. Using UAV-acquired multispectral images, we analyzed reflectance-derived vegetation indices (NDVI, SAVI, and PPR), spectral bands (NIR and red-edge), and gray-level texture metrics across pixel-level and object-level spatial units. Feature importance was systematically evaluated through both linear (Pearson + ANOVA F-score) and nonlinear (RFE + MIC) frameworks, enabling us to identify the features with the highest ecological discriminative capacity for RIFA mound classification.

CNN-based deep learning models, including YOLOv4 and YOLOv5, can effectively perform real-time object localization, particularly in contexts characterized by distinct visual features. Nevertheless, these models generally necessitate extensive volumes of annotated training data and substantial computational resources for competent generalization across variable environments. Although CNN-based models perform well in many detection tasks, they often lack ecological interpretability and struggle to adapt to limited training data. In scenarios with limited data, such as ours, simpler models with selected features offer practical advantages: data are easier to train, require fewer computational resources, and provide enhanced interpretability in their decision-making processes.

While texture and other spatial features did not emerge as primary contributors in our feature selection, this remains promising for more complex non-linear classifiers. These features enhance detection performance when employed within advanced frameworks. For example, augmenting a CNN-based model with explicit texture descriptors, such as Local Binary Pattern (LBP)-based texture images, has demonstrated improvements in classification accuracy [52]. This suggests that integrating texture and similar high-level features in future model enhancements or hybrid methodologies could substantially improve performance.

The framework for feature selection and classification proposed herein offers two critical advantages. First, this framework incorporates biologically based reflectance indicators, such as the NDVI, NIR band, and PPR, that are correlated with ecological characteristics, such as exposed soil and plant stress. Second, the causal exploration enabled by our framework facilitates the identification of interpretable features that connect observable spectral patterns to biophysical conditions. This approach represents an ecologically relevant application of general principles, offering a robust pathway for automated monitoring of ant mounds.

## 5. Conclusions

This research illustrates the value of UAV-based multispectral imaging as an efficient and accurate method for RIFA mound identification in Taiwan, facilitating efficient management of invasive species. Vegetation indices (NDVI, SAVI, and PPR) and spectral features (NIR) were the principal predictors identified through robust feature selection capable of extracting ecological signals regarding low vegetation cover and exposed ground. Although texture features were minimally informative in linear models, they improved nonlinear models by providing intricate spatial patterns. This research provides a basis for the development of an automated model for RIFA mound detection that incorporates the NDVI, NIR, SAVI, and PPR as key features. Our findings are applicable to scalable RIFA monitoring protocols that can be used in larger-scale pest and conservation biology. Additional research is required to integrate hyperspectral sensing, real-time computing, and ground-based methods to improve approaches to efficient, area-wide management of established RIFA colonies to avert the ecological and economic destruction caused by this invasive species.

## Figures and Tables

**Table 1:** Summary of spectral, vegetation, and texture features used in this study. Vegetation indices and texture metrics are derived from UAV-based multispectral imagery. Each feature is accompanied by its calculation method and ecological or analytical role in RIFA mound detection.

Feature Name	Feature Type	Definition	Calculation
NDVI	Vegetation Index	Measures vegetation vigor according to the normalized difference between NIR and red reflectance	$NDVI=\frac{NIR-R}{NIR+R}$
SAVI	Vegetation Index	Adjusts NDVI to account for soil background effects on the basis of a soil brightness correction factor	$SAVI=\frac{\left(NIR-R\right)\times (1+0.5)}{\left(NIR+R+0.5\right)}$
PPR	Vegetation Index	Measures the relative levels of chlorophyll to other plant pigments, which indicate vegetation health, vigor, and potential issues like weed infestation or nutrient stress	$PPR=\frac{Green-Blue}{Green+Blue}$
Homogeneity	Texture Index (GLCM)	Measures local uniformity	$\sum_{i=0}^{N_{g}-1}\sum_{j=0}^{N_{g}-1}\frac{g\left(i,j\right)}{1+{\left(i-j\right)}^{2}}$
Contrast	Texture Index (GLCM)	Measures local variation	$\sum_{i=0}^{N_{g}-1}\sum_{j=0}^{N_{g}-1}{\left(i-j\right)}^{2}\cdot g\left(i,j\right)$
Dissimilarity	Texture Index (GLCM)	Measures gray-level differences between pixel pairs	$\sum_{i=0}^{N_{g}-1}\sum_{j=0}^{N_{g}-1}\left|i-j\right|\cdot g\left(i,j\right)$
Entropy	Texture Index (GLCM)	Measures randomness in image texture	$-\sum_{i=0}^{N_{g}-1}\sum_{j=0}^{N_{g}-1}g\left(i,j\right)\cdot \ln\left(g\left(i,j\right)\right)$
Second Moment	Texture Index (GLCM)	Measures textural smoothness	$\sum_{i=0}^{N_{g}-1}\sum_{j=0}^{N_{g}-1}g{\left(i,j\right)}^{2}$
Correlation	Texture Index (GLCM)	Measures the linear dependency of pixel pairs	$\frac{\sum_{i=0}^{N_{g}-1}\sum_{j=0}^{N_{g}-1}\left(i-\mu \right)\left(j-\mu \right)\cdot g\left(i,j\right)}{\sigma^{2}}$

**Table 2:** Top 10 features ranked by average weighted score obtained through four evaluation methods.

Pixal_Linear	Pixal_ NonLinear	Object_Linear	Object_NonLinear
NDVI	NDVI	SAVI	NIR
Blue	PPR	NIR	PPR
Rededge	NIR	Red	Red
NIR (Entrop)	Blue	RedEdge (Second)	NIR (Contra)
RedEdge (Second)	NIR (Entrop)	Green (Dissim)	Green (Second)
Green (Entrop)	RedEdge (Entrop)	Blue (Second)	Red (Dissim)
Red (Entrop)	Green (Entrop)	RedEdge (Correl)	RedEdge (Correl)
RedEdge (Dissim)	Blue (Entropy)	NIR (Correl)	NIR (Correl)
Blue (Entropy)	RedEdge (Correl)	Green (Correl)	Blue (Correlat)
Blue (Contrast)	Red (Second)	Red (Correl)	Red (Correl)

**Figure A1:**
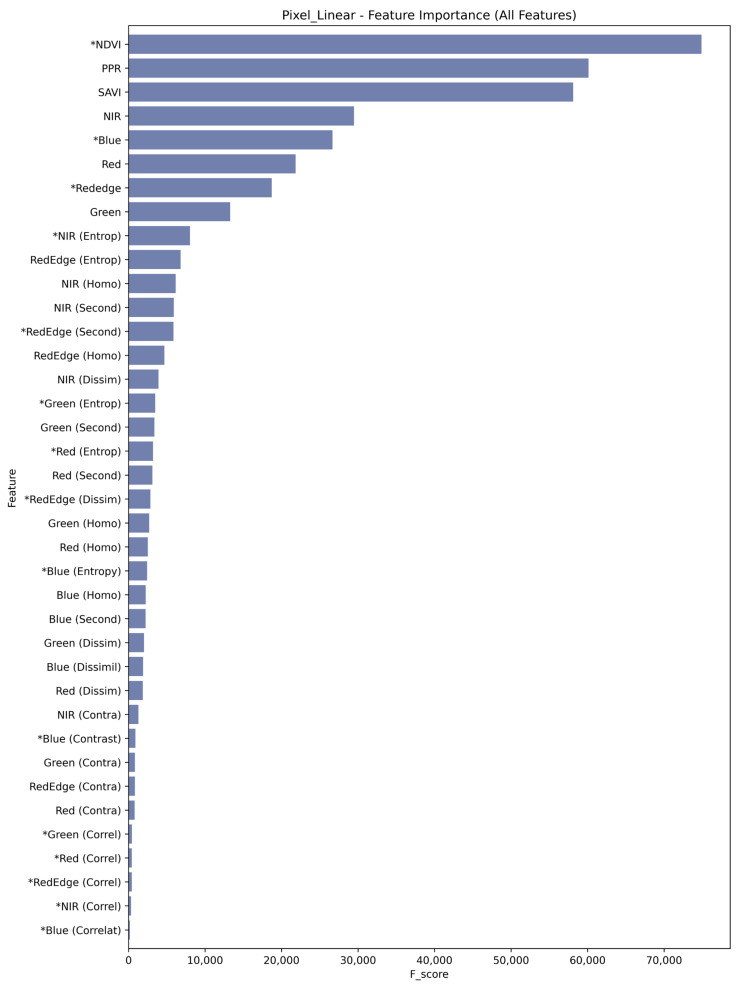
Feature importance ranking within linear pixel-level dataset based on ANOVA F-scores. Vegetation indices, such as the NDVI, PPR, and SAVI, exhibited the highest discriminative power for classifying the presence of RIFA mounds. Spectral bands (NIR, blue) also exhibited high F-scores, supporting their relevance in spectral contrast between mounds and background pixels. Asterisk (*) indicates whether a feature was retained under a given method.

**Figure A2:**
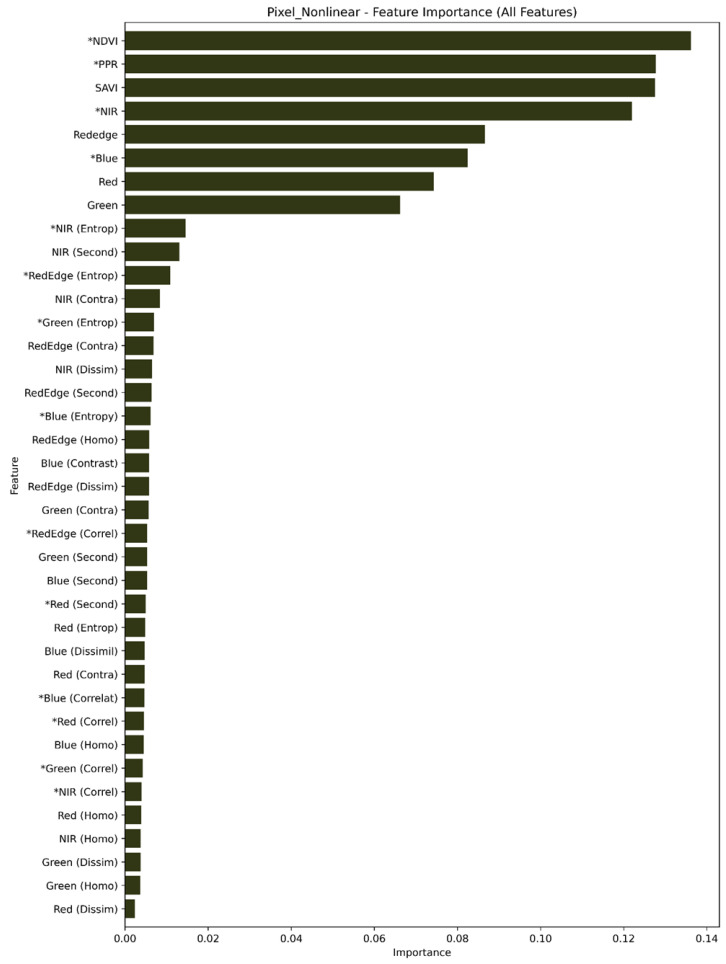
Feature importance ranking within pixel-level nonlinear dataset based on random forest RFE results. Vegetation indices (NDVI, PPR, and SAVI) exhibited the highest importance, highlighting their strong nonlinear separability in pixel-level classification tasks. Primary spectral bands (NIR, blue, and red) and red-edge bands also exhibited high relevance, reinforcing the utility of reflectance-based features. Asterisk (*) indicates whether a feature was retained under a given method.

**Figure A3:**
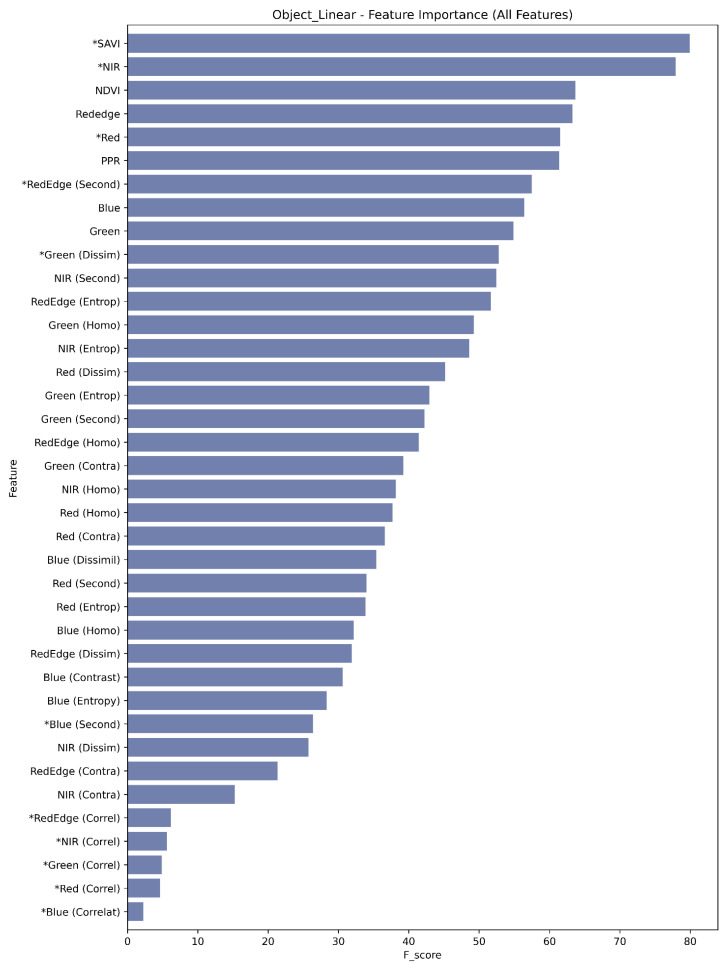
Feature importance ranking within linear object-level dataset based on ANOVA F-scores. Bar chart displays the discriminative power of each feature in classifying the presence of RIFA mounds. Spectral indices, such as the SAVI, NIR, and NDVI, demonstrated the highest F-scores, indicating strong statistical separation between classes. Asterisk (*) indicates whether a feature was retained under a given method.

**Figure A4:**
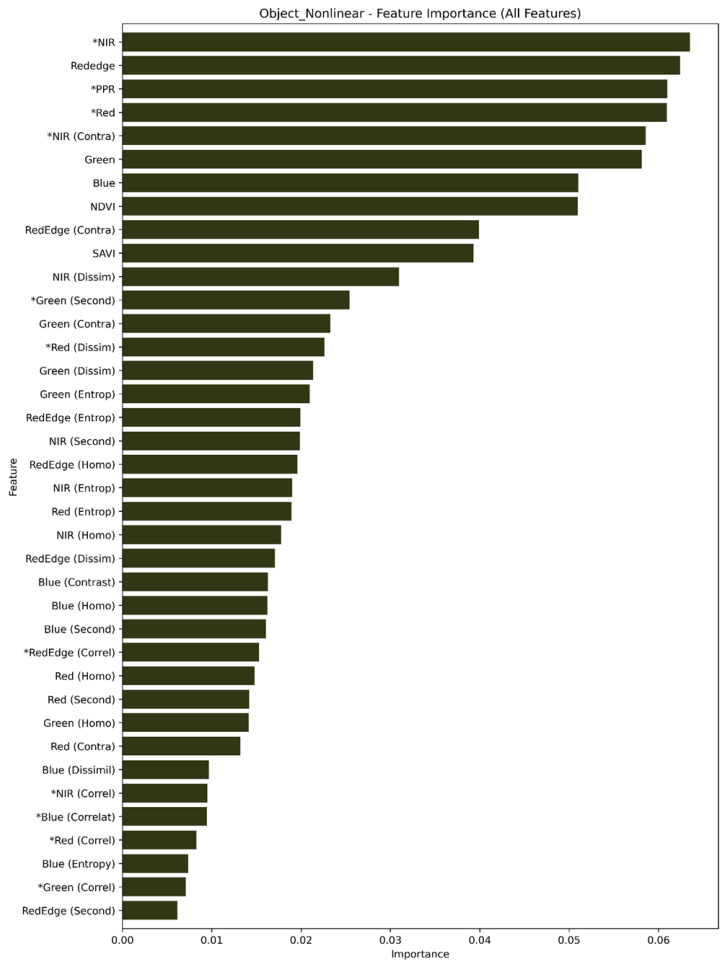
Feature importance ranking within nonlinear object-level dataset based on random forest RFE results. Bar chart displays the relative importance scores of all features regarding nonlinear classification. Primary reflectance band (NIR and red) and spectral indices (PPR) exhibited the highest importance scores, suggesting their dominant role in RIFA mound discrimination. Asterisk (*) indicates whether a feature was retained under a given method.

## Data Availability

The datasets generated during the current study are available from the corresponding author on reasonable request. Restrictions apply because the imagery contains precise farmland locations owned by private landholders and is subject to data-sharing agreements with the Owner.

## Abbreviations

The following abbreviations are used in this manuscript:

ANOVA	Analysis of variance
CNN	Convolutional neural network
GLCM	Gray-level co-occurrence matrix
GNSS	Global navigation satellite system
MIC	Maximal information coefficient
NDVI	Normalized difference vegetation index
NIR	Near-infrared
P4M	Phantom 4 Multispectral Tool
PPR	Photochemical pigment reflectance index
RFE	Recursive feature elimination
RIFA	Red imported fire ant
RGB	Red–green–blue
ROI	Region of interest
RTK	Real-time kinematic
SAVI	Soil-adjusted vegetation index
UAV	Unmanned aerial vehicle
VRS-RTK	Virtual reference station real-time kinematic
YOLO	You only look once
